# miR-10a inhibits cell proliferation and promotes cell apoptosis by targeting BCL6 in diffuse large B-cell lymphoma

**DOI:** 10.1007/s13238-016-0316-z

**Published:** 2016-11-04

**Authors:** Qian Fan, Xiangrui Meng, Hongwei Liang, Huilai Zhang, Xianming Liu, Lanfang Li, Wei Li, Wu Sun, Haiyang Zhang, Ke Zen, Chen-Yu Zhang, Zhen Zhou, Xi Chen, Yi Ba

**Affiliations:** 1 Department of Lymphoma, Sino-US Center for Lymphoma and Leukemia, Tianjin Medical University Cancer Institute and Hospital, National Clinical Research Center of Cancer, Key Laboratory of Cancer Prevention and Therapy, Tianjin, 300060 China; 2 State Key Laboratory of Pharmaceutical Biotechnology, NJU Advanced Institute of Life Sciences, Jiangsu Engineering Research Center for MicroRNA Biology and Biotechnology, School of Life Sciences, Nanjing University, Nanjing, 210093 China; 3 Department of Digestion, Tianjin Medical University Cancer Institute and Hospital, National Clinical Research Center of Cancer, Key Laboratory of Cancer Prevention and Therapy, Tianjin, 300060 China

**Keywords:** microRNA, miR-10a, BCL6, DLBCL, proliferation, apoptosis

## Abstract

The BCL6 (B-Cell Lymphoma 6) gene is a proto-oncogene that is often expressed in diffuse large B-cell lymphomas (DLBCLs). BCL6 loss of function can kill DLBCL cells, demonstrating that BCL6 is necessary for the survival of DLBCL cells and could be a therapeutic target. In this study, we found that BCL6 protein levels were consistently upregulated in DLBCL tissues, whereas its mRNA levels varied randomly in tissues, suggesting that a post-transcriptional mechanism was involved in BCL6 regulation. We used bioinformatics analysis to search for miRNAs, which potentially target BCL6, and identified specific targeting sites for miR-10a in the 3′-untranslated region (3′-UTR) of BCL6. We further identified an inverse correlation between miR-10a levels and BCL6 protein levels, but not mRNA levels, in DLBCL tumor tissue samples. By overexpressing or knocking down miR-10a in DLBCL cells, we experimentally validated that miR-10a directly recognizes the 3′-UTR of the BCL6 transcript and regulated BCL6 expression. Furthermore, we demonstrated that negatively regulating BCL6 by miR-10a suppressed the proliferation and promoted apoptosis of DLBCL cells.

**Electronic supplementary material:**

The online version of this article (doi:10.1007/s13238-016-0316-z) contains supplementary material, which is available to authorized users.

## Introduction

Worldwide, diffuse large B-cell lymphoma (DLBCL) is the most common type of lymphoma, accounting for 30%–40% of newly diagnosed non-Hodgkin lymphoma (NHL) cases (Yatomi, 2012). Enormous progress has been made in therapy, and the average five-year overall survival is approximately 50% (Gerrard et al., 2013). Despite improvements in the treatments, DLBCL is still associated with a high mortality rate (Westin and Fayad, 2009); approximately one-third of patients with DLBCL will be refractory to therapy or relapse (Van Den Neste et al., 2015).

The BCL6 (B-Cell Lymphoma 6) gene is a member of the BTB-POZ family and is the most frequently involved oncogene in DLBCL (Parekh et al., 2007). Recent studies have demonstrated that BCL6 plays an important role in the formation of germinal center (GC) B cells, which are the cells of origin of DLBCLs (Basso and Dalla-Favera, 2012; Ding et al., 2015). BCL6 can impact DLBCL through modulating B-cell activation, differentiation, cell cycle arrest and apoptosis (Polo et al., 2004; Shaffer et al., 2000). In addition, BCL6 is involved in the development of CD4+ T-follicular helper cells that play a critical role during the generation of germinal centers (Yu et al., 2009; Hollister et al., 2013). Thus, most B cell lymphomas arise from GC B cells need continued expression of BCL6 to maintain their survival (Hatzi et al., 2013; Bertolo et al., 2013). Targeted inhibition of these BCL6 functions has emerged as the basis for the rational design of lymphoma therapies and combinatorial regimens.

MicroRNAs (miRNAs) are a class of 19-24-nucleotide-long short noncoding RNAs, which regulate genes in a sequence-specific manner (Krol et al., 2010; Koscianska and Krzyzosiak, 2014). They play key roles in regulating the translation and degradation of mRNAs by antisense complementarity to specific mRNA, resulting in either direct RNA degradation or inhibition of protein translation (Koscianska and Krzyzosiak, 2014; Pillai et al., 2007). Although the biological functions of most miRNAs are not yet fully understood, it has been suggested that they are involved in various biological processes, including cell proliferation, cell death, stress resistance, and fat metabolism, through regulation of gene expression (Ivey and Srivastava, 2015). Increasing evidence has indicated that miRNAs, in fact, may be key regulators of various fundamental biological processes. In the past years, many research groups have focused on the potential clinical application of microRNAs as diagnostic or therapeutic tools for patients with DLBCL (Jung and Aguiar, 2009; Wang et al., 2014). Some papers have reported the deregulation of miR-10 family members in several human cancers (Khan et al., 2015; Zeng and Li, 2014), such as gastric, bladder, cervical and thyroid cancer. *Anja* and colleagues found that miR-10a is downregulated in hematological tumor cell lines (Agirre et al., 2008), and miR-10a was reported to be downregulated in DLBCL (Roehle et al., 2008). Early studies indicated that miR-10a could regulate the development and activation of immunocytes by targeting BCL6 and its co-repressor Ncor2, which impacts the stability of the differentiation of Tregs (Takahashi et al., 2012).

Although the dysregulation of miR-10a and BCL6 plays an important role in immunoregulation, no correlation between BCL6 and miR-10a in DLBCL has been reported. In this study, we predicted that BCL6 is a target of miR-10a. After measuring the expression levels of miR-10a and BCL6 in human DLBCL tumor tissues and paired non-neoplastic lymphatic tissues, we confirmed an inverse correlation between miR-10a and the BCL6 protein levels. Furthermore, we experimentally validated the direct inhibition of BCL6 translation by miR-10a through overexpressing or knocking down miR-10a in DLBCL cell lines. Finally, we showed the direct regulation of BCL6 by miR-10a and the biological role of miR-10a targeting BCL6 in human DLBCL.

## Results

### Upregulation of BCL6 protein, but not mRNA, in DLBCL tissues

**Figure 1:**
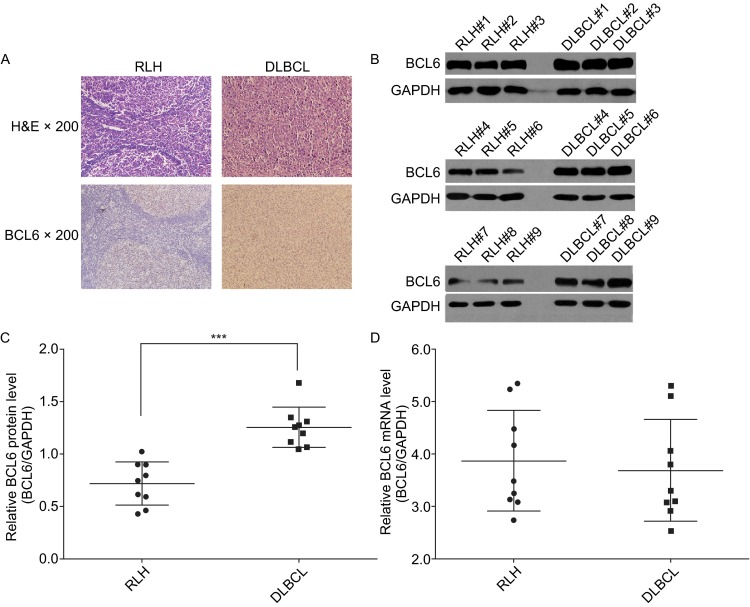
**BCL6 protein and mRNA in human tissues**. (A) Representative H&E-stained and BCL6-stained sections of the DLBCL&RLH tissues; Western blotting analysis of the expression levels of BCL6 protein in 9 cases of DLBCL and 9 cases of RLH. (B) Representative image. (C) Quantitative analysis; (D) Quantitative RT-PCR analysis of BCL6 mRNA levels in the same DLBCL and RLH tissues, the relative expression was assessed using ΔCt values (ΔCt = Ct_BCL6_ − Ct_GAPDH_). The *GAPDH* gene served as the endogenous control. Data (mean ± SEM) are representative of 3 technique replicates. *** *P* < 0.001

The diffuse large B-cell lymphomas (DLBCL) and reactive lymph node hyperplasia (RLH) tissues were embedded in paraffin and then stained with H&E or immunohistochemical staining of Bcl6 for histology examination (Fig. 1A). After measuring the levels of BCL6 protein in DLBCL and RLH tissues via Western blotting, we found that BCL6 protein levels were significantly higher in the DLBCL tissues (Fig.1B, C). Subsequently, we performed quantitative RT-PCR to measure the levels of BCL6 mRNA in the same DLBCL and RLH tissues (Fig. 1D). We found that BCL6 mRNA and protein levels did not correlate between the DLBCL and RLH tissues (Fig. S1). This disparity between the BCL6 protein and mRNA levels in DLBCL tissues strongly suggests that a post-transcriptional mechanism is involved in the regulation of BCL6.

### Identification of conserved miR-10a target sites within the 3′-UTR of BCL6

**Figure 2:**
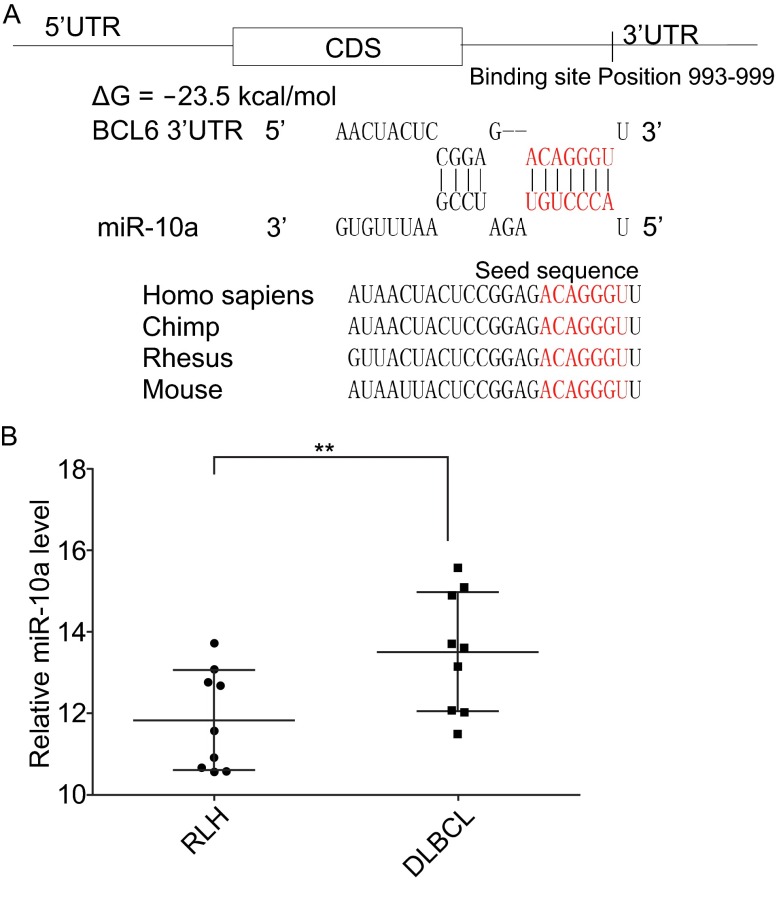
**Schematic description of the hypothesized and miR-10a in human tissues**. (A) Schematic description of the hypothesized duplex formed by interaction between the BCL6 3′-UTR (top) binding site and miR-10a (bottom). The predicted free energy value of the hybrid is indicated. The seed recognition site is denoted, and all nucleotides in this region are highly conserved across species, including Homo sapiens, chimp, rhesus, and mouse. (B) Quantitative RT-PCR analysis of the expression levels of miR-10a, the relative expression levels were analyzed using ΔCt values (ΔCt = Ct_miR-10a_ − Ct_U6_). The *U6* gene served as the endogenous control in diffuse large B-cell lymphoma(DLBCL) and reactive lymph node hyperplasia (RLH) tissues. Data (mean ± SEM) are representative of 3 technique replicates. ** *P* < 0.01

One important mode of post-transcriptional regulation is the repression of mRNA transcripts by miRNAs. miRNAs are therefore likely to play a biologically relevant role in regulating BCL6 expression in DLBCL. Three computational algorithms, including TargetScan (Lewis et al., 2003), miRanda (John et al., 2004) and PicTar (Krek et al., 2005), were used in combination to identify potential miRNAs that can target BCL6. Using these approaches, miR-10a was identified as a candidate regulator of BCL6. The predicted interactions between miR-10a and the targeting sites within the 3′-UTR of BCL6 are illustrated in Fig. 2A. One predicted hybridization was observed between miR-10a and the 3′-UTR of BCL6. There was perfect complementarity between the seed region (the core sequence that encompasses the first 2–8 bases of the mature miRNA) and the putative target sequence. The minimum free energy value of the hybridization between miR-10a and BCL6 was −23.5 kcal/mol, which is well within the range of genuine miRNA-target pairs. Furthermore, the miR-10a binding sequences in the BCL6 3′-UTR were highly conserved across species. Thus, miR-10a was selected for further experimental verification of its binding to BCL6.

### Identification of an inverse correlation between the miR-10a and BCL6 levels in DLBCL tissues

miRNAs are generally thought to have expression patterns that are opposite to those of their targets (Ambros, 2004; Bartel, 2004; He and Hannon, 2004). We next investigated whether miR-10a was inversely correlated with BCL6 in DLBCL. After determining the levels of miR-10a in the same DLBCL and RLH tissues, we found that the miR-10a levels were indeed downregulated in DLBCL tissues (Fig. 2B). Combining the computational prediction with the detection of an inverse correlation between miR-10a and BCL6 *in vivo*, it is quite likely that miR-10a is involved in the post-transcriptional regulation of BCL6.

### Validation of BCL6 as a direct target of miR-10a

**Figure 3:**
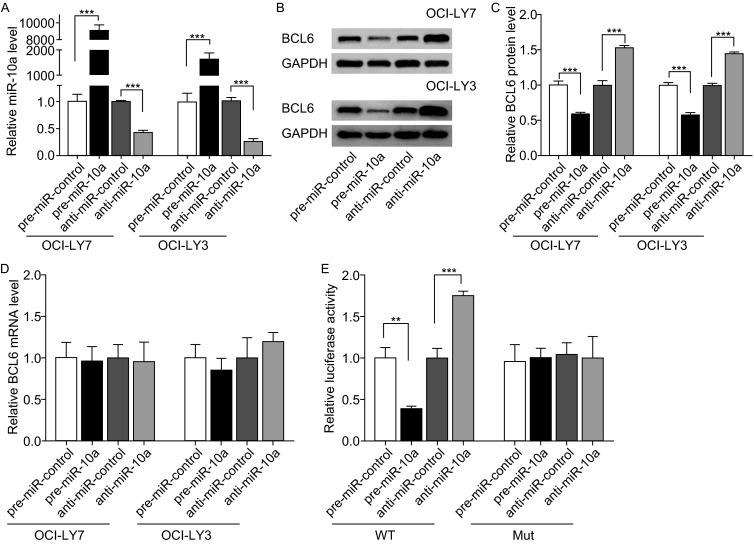
**Direct regulation of BCL6 expression by miR-10a at the posttranscriptional level**. (A) Quantitative RT-PCR analysis of miR-10a levels in OCI-LY7 and OCI-LY3 cells treated with pre-miR-control, pre-miR-10a, anti-miR-control or anti-miR-10a. U6 snRNA was used as an internal control, and the relative amount of miRNA normalized to the U6 snRNA levels was calculated using the 2^-ΔΔCT^ formula, in which ΔΔC_T_ = (C_T miRNA_ - C_T U6_) _target_ - (C_T miRNA_ - C_T U6_) _control_. (B and C) Western blot analysis of BCL6 protein levels in OCI-LY7 and OCI-LY3 cells treated with pre-miR-control, pre-miR-10a, anti-miR-control or anti-miR-10a. B: representative image. C: quantitative analysis. (D) Quantitative RT-PCR analysis of BCL6 mRNA levels in OCI-LY7 and OCI-LY3 cells treated with pre-miR-control, pre-miR-10a, anti-miR-control or anti-miR-10a. (E) Direct recognition of the BCL6 3′-UTR by miR-10a. HEK293T cells were co-transfected with firefly luciferase reporters containing either wild-type (WT) or mutant (MUT) miR-10a binding sites in the BCL6 3′-UTR and pre-miR-control, pre-miR-10a, anti-miR-control or anti-miR-10a, 24 h after transfection, the cells were assayed using a luciferase assay kit. Data are the mean±SEM of 3 independent experiments performed in triplicate, ** *P* < 0.01; *** *P* < 0.001

The correlation between miR-10a and BCL6 expression was further examined by evaluating BCL6 expression in the human DLBCL cell lines OCI-LY7 and OCI-LY3 after the knockdown and overexpression of miR-10a. In these experiments, miR-10a overexpression was achieved by transfecting OCI-LY7 and OCI-LY3 cells with pre-miR-10a (a synthetic RNA oligonucleotide that mimics the miR-10a precursor); and miR-10a knockdown was achieved by transfecting cells with anti-miR-10a, (a chemically modified antisense oligonucleotide designed to specifically target mature miR-10a). As anticipated, cellular miR-10a levels were significantly increased when OCI-LY7 and OCI-LY3 cells were transfected with pre-miR-10a; and were decreased when OCI-LY7 and OCI-LY3 cells were treated with anti-miR-10a (Fig. 3A). The expression of the BCL6 protein was reduced by the overexpression of miR-10a and increased by the knockdown of miR-10a in OCI-LY7 and OCI-LY3 cells (Fig. 3B and 3C). The expression of BCL6 protein was significantly inhibited by the introduction of pre-miR-10a into OCI-LY7 and OCI-LY3 cells, while anti-miR-10a significantly increased the BCL6 protein level in OCI-LY7 and OCI-LY3 cells. To determine the level at which miR-10a regulates BCL6 expression, we repeated the above experiments and examined the expression of BCL6 mRNA after transfection. The overexpression or knockdown of miR-10a did not affect the mRNA stability of BCL6 (Fig. 3D). These results demonstrated that miR-10a specifically regulated BCL6 protein expression at the post-transcriptional level, which is the most common mechanism of animal miRNA action.

To determine whether the negative regulatory effects of miR-10a on BCL6 expression were mediated by the binding of miR-10a to the predicted target sites in the 3′-UTR of the BCL6 mRNA, the full-length 3′-UTR of BCL6 containing the predicted miR-10a binding site was inserted downstream of the firefly luciferase gene in a reporter plasmid. After we proved miR-10a is expressed in HEK293T cells (Fig. S2), the resulting plasmid was co-transfected into HEK293T cells with a transfection control plasmid (β-gal) and either pre-miR-10a or anti-miR-10a. As expected, the luciferase activity was markedly reduced in the cells transfected with pre-miR-10a, whereas the inhibition of miR-10a resulted in an increase in reporter activity compared with transfection with anti-miR control (Fig. 3E). Furthermore, we introduced point mutations into the corresponding complementary sites in the 3′-UTR of BCL6 to disrupt the predicted miR-10a binding sites. This mutated luciferase reporter was unaffected by the overexpression of miR-10a (Fig. 3E). This finding suggested that the binding sites of BCL6 strongly contributed to this miRNA-mRNA interaction, which mediates the post-transcriptional repression of BCL6 expression. In conclusion, our results demonstrate that miR-10a directly binds to the 3′-UTR of the BCL6 mRNA transcript to suppress BCL6 expression.

### miR-10a inhibits proliferation and promotes the apoptosis of DLBCL cells by targeting BCL6

We next analyzed the biological consequences of the miR-10a-driven repression of BCL6 expression in DLBCL cells. Because BCL6 is known to be required for DLBCLs to maintain their proliferate and survival (Parekh et al., 2008), we investigated whether the knockdown or overexpression of miR-10a or BCL6 would impact the cell cycle, proliferation and apoptosis of OCI-LY7 cells.

To knock down BCL6 expression, the siRNA sequence targeting different sites of human BCL6 cDNA was designed and transfected into OCI-LY7 cells. To overexpress BCL6, a plasmid expressing the BCL6 ORF was transfected into OCI-LY7 cells. The efficient knockdown or overexpression of BCL6 is demonstrated in Supplementary Figure 3. And the BCL6 protein levels in the samples treated with pre- miR10a or rescued with BCL6 cDNA is demonstrated in Supplementary Figure 4.

**Figure 4:**
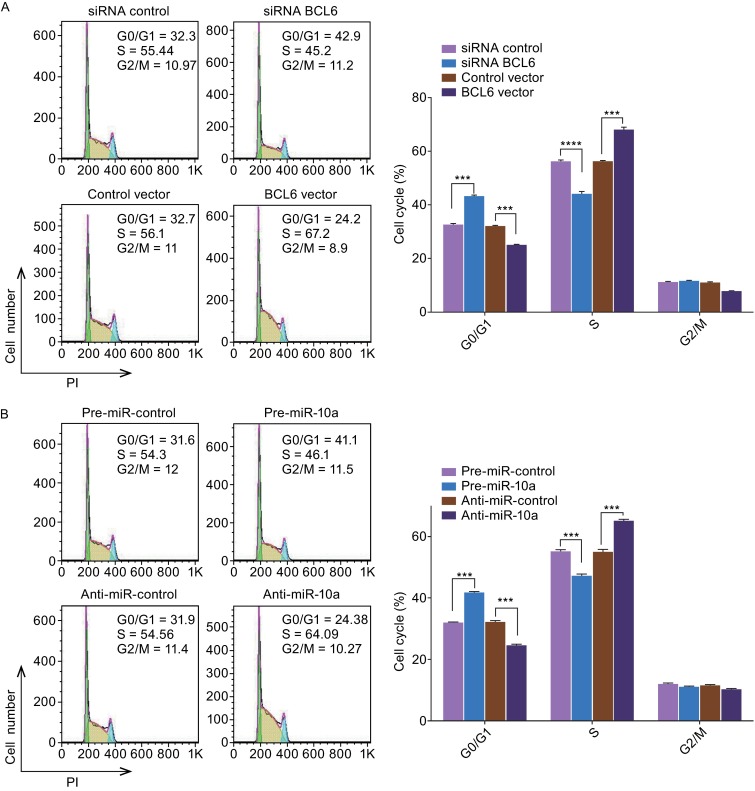

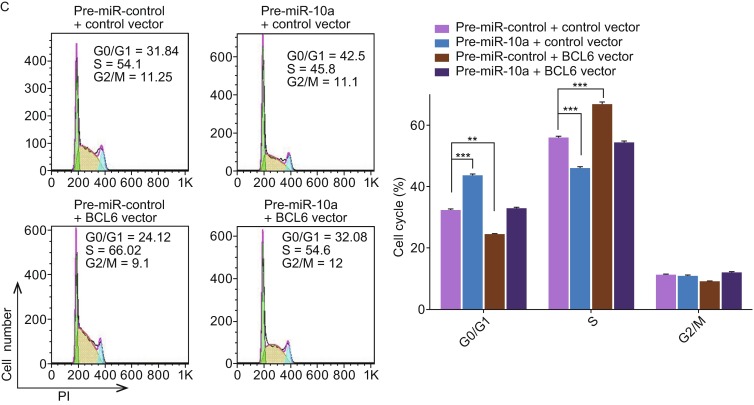
**The role of miR-10a targeting BCL6 in the regulation of cell cycle in DLBCL cells**. (A) Cell cycle profiles were analyzed using flow cytometry afer the transfection of OCI-LY7 cells with equal dose of control siRNA, BCL6 siRNA, control vector or BCL6 overexpression vector. (B) Cell cycle profiles were analyzed using flow cytometry afer the transfection of OCI-LY7 cells with equal dose of pre-miR-control, pre-miR-10a, anti-miR-control or anti-miR-10a. (C) Cell cycle profiles were analyzed using flow cytometry afer the transfection of OCI-LY7 cells with pre-miR-control plus control vector, a pre-miR-control plus BCL6 overexpression vector, pre-miR-10a plus control vector, or pre-miR-10a plus BCL6 overexpression vector. The panel shows hisograms of cell numbers (y axis) against DNA content (x axis) determined by measuring fluorescence intensity. Numbers denote the percentages of cells in the G0/G1, S and G2/M phases. Left panel: representative image; right panel: quantitative analysis. Data are the mean ± SEM of 3 independent experiments performed in triplicate, ** *P* < 0.01; *** *P* < 0.001

**Figure 5:**
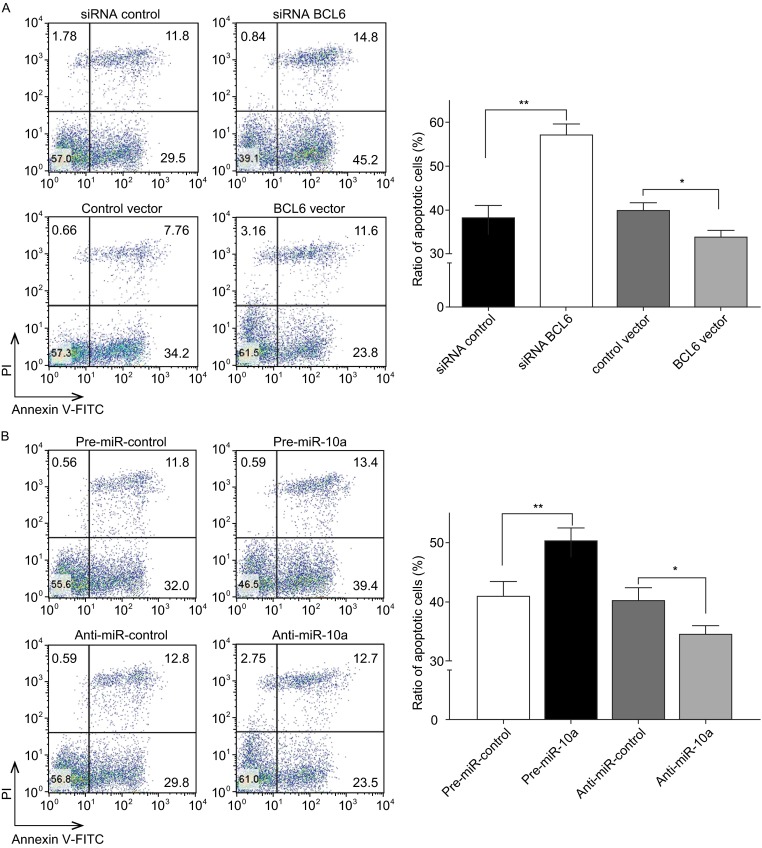

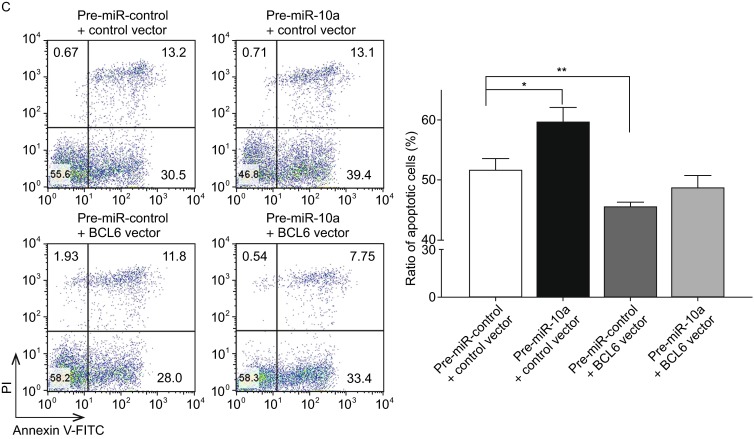
**The role of miR-10a targeting BCL6 in the regulation of apoptosis in DLBCL cells**. (A)The apoptosis assay was performed 24 h after the transfection of OCI-LY7 cells with equal dose of control siRNA, BCL6 siRNA, control vector or BCL6 overexpression vector; (B) The apoptosis assay was performed 24h after the transfection of OCI-LY7 cells with equal dose of pre-miR-control, pre-miR-10a, anti-miR-control or anti-miR-10a; (C)The apoptosis assay was performed 24h after the transfection of OCI-LY7 cells with pre-miR-control plus control vector, a pre-miR-control plus BCL6 overexpression vector, pre-miR-10a plus control vector, or pre-miR-10a plus BCL6 overexpression vector. Cell apoptosis profiles were analyzed by flow cytometry. The biparametric histogram shows cells in early (bottom right quadrant) and late apoptotic states (upper right quadrant). Viable cells are double negative (bottom left quadrant). Left panel: representative image; right panel: ratio of apoptotic OCI-LY7 cells. Data are the mean ± SEM of 3 independent experiments performed in triplicate, * *P* < 0.05; ** *P* < 0.01

**Figure 6:**
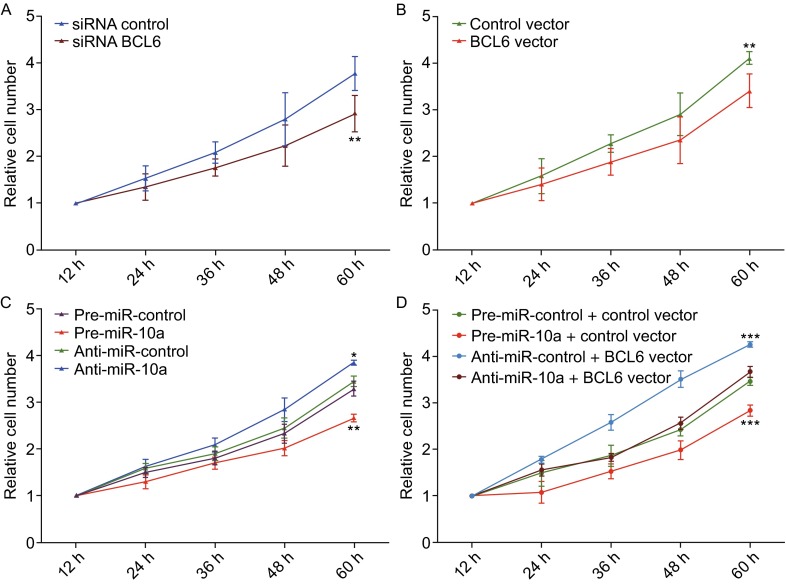
**The role of miR-10a targeting BCL6 in the regulation of proliferation in DLBCL cells**. (A) CCK8 viability assays were performed 12 h, 24 h, 36 h, 48 h and 60 h after the transfection of OCI-LY7 cells with either a control siRNA or a BCL6 siRNA. (B) CCK8 viability assays were performed 12 h, 24 h, 36 h, 48 h and 60 h after the transfection of OCI-LY7 cells with either a control vector or a BCL6 overexpression vector. (C) CCK-8 viability assays were performed 12 h, 24 h, 36 h, 48 h and 60 h after the transfection of OCI-LY7 cells with pre-miR-control, pre-miR-10a, anti-miR-control or anti-miR-10a. (D) CCK-8 viability assays were performed 12 h, 24 h, 36 h, 48 h and 60 h after the transfection of OCI-LY7 cells with a pre-miR-control plus control vector, pre-miR-10a plus control vector, a pre-miR-control plus BCL6 overexpression vector, or pre-miR-10a plus BCL6 overexpression vector. Data are the mean ± SEM of 3 independent experiments performed in triplicate, * *P* < 0.05; ** *P* < 0.01; *** *P* < 0.001

The proliferation of OCI-LY7 cells was examined using the cell cycle and the cck8 assay. OCI-LY7 cells transfected with the BCL6 siRNA resulted in the initial accumulation of cells in G0/G1-phase of the cell cycle, in contrast, the OCI-LY7 cells transfected with the BCL6 overexpression plasmid showed a reduction of cells in the G0/G1-phase, whereas the numbers of cells in the S phases increased (Fig. 4A). And OCI-LY7 cells with BCL6 knocked down using siRNA exhibited the promotion of apoptosis, the cells transfected with the BCL6 overexpression plasmid exhibited significantly reduced apoptosis (Fig. 5A). Meanwhile, We evaluated the collective effects of BCL6 on the growth and survival of OCI-LY7 cells using the CCK8 assay. The knockdown of BCL6 inhibited the growth and survival of OCI-LY7 cells; by contrast, to overexpress BCL6 had the opposite effect on the growth and survival of OCI-LY7 cells (Fig. 6A and 6B).

Subsequently, we analyzed the biological consequences of the miR-10a-mediated suppression of BCL6 expression in DLBCL cells. OCI-LY7 cells transfected with pre-miR-10a exhibited G0/G1-phase arrest; by contrast, knocking down of miR-10a the numbers of cells in the S phases increased (Fig. 4B). Moreover, compared with the cells transfected with pre-miR-10a, the cells co-transfected with pre-miR-10a and the BCL6 overexpression plasmid exhibited reduction of cells in the G0/G1-phase, and the numbers of cells in the S phases increased (Fig. 4C), suggesting that miR-10a-resistant BCL6 expression rescued the suppression of BCL6 expression by miR-10a and attenuated the effect on cell cycle of miR-10a. We investigated the effects of miR-10a on DLBCL cell apoptosis via flow cytometer. The percentage of apoptotic cells was higher among the OCI-LY7 cells transfected with pre-miR-10a but was lower among the OCI-LY7 cells transfected with anti-miR-10a (Fig. 5B). Furthermore, when OCI-LY7 cells were simultaneously transfected with pre-miR-10a and the BCL6 overexpression plasmid, the pro-apoptotic effect of miR-10a was dramatically attenuated (Fig. 5C). Finally, we analyzed the collective effects of miR-10a-mediated suppression of BCL6 expression on the growth and survival of DLBCL cells using the CCK8 assay. OCI-LY7 cells transfected with pre-miR-10a suppressed the growth and survival of OCI-LY7 cells; by contrast, knockdown of miR-10a had the opposite effect on the growth and survival of OCI-LY7 cells (Fig. 6C). Moreover, compared with the cells transfected with pre-miR-10a, the cells co-transfected with pre-miR-10a and the BCL6 overexpression plasmid attenuated the inhibition effect of the growth and survival induced by miR-10a (Fig. 6D), suggesting that miR-10a-resistant BCL6 expression rescued the suppression of BCL6 expression by miR-10a. These results indicate that miR-10a might inhibits proliferation and promotes the apoptosis by downregulating BCL6 in DLBCL cells.

## Discussion

BCL6 as a transcriptional repressor, often involved in the development of DLBCL. It is an important gene in B-cell differentiation, which can mediate apoptosis, cell cycle control, survival and inflammatory reaction. BCL6 can influence the prognosis of patients with DLBCL at gene and protein levels and therefore is an independent prognostic factor for DLBCL (Gao et al., 2014; Winter et al., 2006). It is also essential for GC formation (Baron et al., 2004). Thus, most DLBCLs need BCL6 to maintain their survival, and the expression of BCL6 plays a key role in DLBCL. Multiple mechanisms act coordinately to timely modulate BCL6 expression at the transcriptional and post-transcriptional levels (Basso and Dalla-Favera, 2012). BCL6 loss of function can kill DLBCL cells, demonstrating that BCL6 is required for the survival of DLBCL cells and could be an excellent therapeutic target. Recent studies have intended to identify highly specific and non-toxic BCL6 inhibitors (Cerchietti and Melnick, 2013; Duan et al., 2012). Targeting RNA by RNA interference (RNAi) or antisense oligonucleotides (ASOs) has been attempted to eradicate BCL6 from lymphoma cells (Saito et al., 2006). In this study, we found an alternative mechanism—regulating BCL6 expression at the posttranscriptional level in DLBCL.

In this study, we found that silencing BCL6 expression by siRNA could inhibit proliferation and promote apoptosis in DLBCL cells, whereas overexpressing BCL6 induced opposing effects, validating its central role as an essential oncogene during DLBCL tumorigenesis. Interestingly, we identified discordance between the BCL6 protein and mRNA levels in human DLBCL tissues. These results suggested a post-transcriptional regulation mechanism involved in BCL6 repression. One centrally important mode of post-transcriptional regulation is the repression of mRNA transcripts by miRNAs. Therefore, we searched for miRNAs that could target BCL6 and identified miR-10a as a candidate. In addition, by overexpressing or knocking down miR-10a in DLBCL cells, we experimentally validated the direct inhibition of BCL6 translation by miR-10a. Finally, we showed that miR-10a inhibited BCL6 expression resulted in the initial accumulation of cells in G0/G1-phase of cell cycle companied with the inhibited proliferation and promoted apoptosis in cultured DLBCL cells. The results delineate a novel regulatory network employing miR-10a and BCL6 to fine tune cell proliferation and apoptosis. Additionally, a recent study has shown that miR-10a directly targets Bcl-6 to downregulate protein expression level in a murine B cell lymphoma cell line(Takahashi et al., 2012). The latter study, combined with ours, reveals the importance of miR-10a targeting BCL6 as a novel regulatory pathway in DLBCL progression.

miRNAs are aberrantly expressed in cancer and can function as oncogenes or tumor suppressor genes (Calin and Croce, 2006; Esquela-Kerscher and Slack, 2006). In the present study, we found that the levels of miR-10a were lower in DLBCL tumor tissues than in normal adjacent tissues. These results suggest that miR-10a may be involved in the pathogenesis of DLBCL as a tumor suppressor. Indeed, it is reported in many studies that miR-10a is downregulated in cancers (Havelange et al., 2014; Yan et al., 2013). On the other hand, it is well known that a single miRNA can target multiple genes, while multiple miRNAs can target a single gene. Thus, miR-10a may have multiple different mRNA targets other than BCL6, and these additional targets may also play important roles in carcinogenesis. For example, miR-10a was reported to be downregulated in hematological cancer cell lines (Gaur et al., 2007) such as acute myeloid leukemia (Zhang et al., 2006). Therefore, at this stage, the most important question is to investigate how critical this new pathway would be in the field of DLBCL carcinogenesis. During this study, we found that overexpressing miR-10a could inhibit proliferation and promote apoptosis in DLBCL cells and that BCL6 reduction could mimic miR-10a induction. Interestingly, we observed that the restoration of BCL6 expression could successfully attenuate the anti-proliferative and pro-apoptotic effects of miR-10a on DLBCL cells, although miR-10a has many other targets. These results suggest that the targeting of BCL6 is a major mechanism by which miR-10a exerts its tumor-suppressive function. Therefore, the modulation of BCL6 by miR-10a might explain, at least in part, why the downregulation of miR-10a during DLBCL carcinogenesis can promote cell growth and accelerate DLBCL formation.

BCL6 as a pro-oncogenic has emerged as a critical therapeutic target in DLBCL. The biochemical study of BCL6-mediated gene repression has provided the basis for the design of agents that inhibit BCL6 and kill lymphoma cells (Parekh et al., 2008). Recently, functional and biochemical studies have provided the basis and rational for the development of highly specific and nontoxic BCL6 inhibitors (Basso and Dalla-Favera, 2012; Cerchietti and Melnick, 2013). Thus, BCL6 is likely to become a new target for DLBCL treatment. On the other hand, given the dysregulation of miRNAs in cancer development, the correction of cellular miRNA levels has emerged as a potential therapeutic strategy. The overexpressed miRNAs can be silenced using miRNA ASOs, and the re-expression of miRNAs that are lost in cancers can be achieved by overexpressing miRNA mimics. In this study, the results suggest that targeting BCL6 is a major mechanism by which miR-10a exerts its tumor-suppressive and pro-apoptotic function in DLBCL cells. Thus, it is hypothesized that a replacement treatment with miR-10a mimics may be a promising strategy for cancers characterized by miR-10a downregulation. In summary, as important emerging modulators in cellular pathways, miR-10a and BCL6 may provide attractive, novel therapeutic targets for DLBCL treatment. In future studies, treatments with both miR-10a mimics and BCL6-targeted drugs may offer a viable strategy for DLBCL therapy.

Together, the results of this study delineate a novel regulatory network employing miR-10a and BCL6 to fine tune proliferation and apoptosis in DLBCL cells. This study may provide a potential novel target for future DLBCL therapy.

## MATERIALS AND METHODS

### Clinical samples

The patients were eligible if they had previously untreated, biopsy-confirmed diffuse large B-cell lymphoma according to the World Health Organization criteria. In addition, 9 anonymized samples of histopathological verified reactive lymph node hyperplasia (RLH) were included as normal control. The diffuse large B-cell lymphoma tumor tissues and reactive lymph node hyperplasia tissues were derived from patients undergoing a surgical procedure at the Tianjin Medical University Cancer Institute and Hospital (Tianjin, China). Tissue fragments were immediately frozen in liquid nitrogen at the time of surgery and stored at −80°C. The clinical features of the DLBCL patients are listed in Supplementary Table 1.

### Cell culture and reagents

The cell lines OCI-LY7, OCI-LY3 and HEK293T were obtained from American Type Culture Collection (ATCC). OCI-LY7 is germinal center B-cell (GCB)-subtype cell line; OCI-LY3 is an activated B-cell (ABC)-subtype cell line; and HEK293T is an embryonic kidney cell line. OCI-LY7 was maintained in complete Iscove’s modified essential medium (IMDM; GIBCO, Carlsbad, CA, USA) with 2-mercaptoethanol (1:10000) and 10% fetal bovine serum (GIBCO). OCI-LY3 was cultured in IMDM with 20% fetal bovine serum. HEK293T cells were grown in Dulbecco’s Modified Eagle’s Medium (DMEM; GIBCO) supplemented with 10% fetal bovine serum. All of the cell lines were supplemented with 1% penicillin/streptomycin (Invitrogen, Carlsbad, CA, USA). All of the cell cultures were maintained at 37°C under 5% CO_2_ and 95% air.

### RNA isolation and quantitative RT-PCR

Total RNA was extracted from the cultured cells or tissues using Trizol reagent (Invitrogen) according to the manufacturer’s instructions. Assays to quantify mature miRNAs were performed using TaqMan miRNA probes (Applied Biosystems, Foster City, CA, USA) according to the manufacturer’s instructions. Briefly, 1 µg of total RNA was reverse transcribed to cDNA using AMV reverse transcriptase (TaKaRa, Dalian, China) and a stem-loop RT primer (Applied Biosystems). The reaction conditions were as follows: 16°C for 30 min, 42°C for 30 min and 85°C for 5 min. Real-time PCR was performed using a TaqMan PCR kit and an Applied Biosystems 7500 Sequence Detection System (Applied Biosystems). The reactions were incubated in a 96-well optical plate at 95°C for 5 min, followed by 40 cycles of 95°C for 15 s and 60°C for 1 min. All of the reactions were performed in triplicate. After the reactions were complete, the cycle threshold (C_T_) data were collected using fixed threshold settings, and the mean C_T_ was determined from triplicate PCRs. A comparative C_T_ method was used to compare each transcript with the controls. U6 snRNA was used as an internal control, and the relative amount of miRNA normalized to the U6 snRNA levels was calculated using the 2^-ΔΔCT^ formula, in which ΔΔC_T_ = (C_T miRNA_ − C_T U6_) _target_ − (C_T miRNA_ − C_T U6_) _control_.

To quantify the BCL6 and GAPDH mRNA levels, 1 µg of total RNA was reversely transcribed to cDNA using oligod (T) 18 primers (TaKaRa) and ThermoScript reverse transcriptase (Invitrogen). The reaction conditions were as follows: 42°C for 60 min and 70°C for 10 min. Real-time PCR was then performed with the RT product, and these reactions included SYBR Green dye (Invitrogen) and specific primers for BCL6 and GAPDH. The sequences of the primers were as follows: 5′-ACTCCCATGTGATAGTGCCA-3′ (BCL6 sense) and 5′-GTGCCTCTTCTGGGATTGTT-3′ (BCL6 antisense); 5′-GATATTGTTGCCATCAATGAC-3′ (GAPDH sense) and 5′-TTGATTTTGGAGGGATCTCG-3′ (GAPDH antisense). The reactions were incubated at 95°C for 5 min, followed by 40 cycles of 95°C for 30 s, 60°C for 30 s and 72°C for 30 s. After the reactions were completed, the C_T_ values were determined by setting a fixed threshold. The relative amount of BCL6 mRNA was normalized to that of GAPDH.

### Overexpression and knockdown of miR-10a

miRNA overexpression was achieved by transfecting cells with a pre-miR-10a (a synthetic RNA oligonucleotide duplex mimicking the miRNA precursor), whereas knockdown was achieved by transfecting cells with an anti-miR-10a (a chemically modified single-stranded antisense oligonucleotide designed to specifically target mature miRNA). Synthetic pre-miR-10a, anti-miR-10a, pre-miR-control and anti-miR-control RNAs were purchased from Genepharma (Shanghai, China). Next, 3 × 10^6^ OCI-LY7 or OCI-LY3 cells were seeded per well in 6-well plates and were transfected with Lipofectamine 2000 (Invitrogen) following the manufacturer’s protocols. The cells were harvested 24 h or 48 h after transfection for quantitative RT-PCR or Western blotting. The transfection efficiency was determined by quantitative RT-PCR for hsa-miR-10a. Transfection experiments were repeated three times independently and in each case were done in triplicate.

### miRNA target prediction

The miRNAs that may target BCL6 were determined using algorithms from TargetScan (http://genes.mit.edu/targetscan/), PicTar (http://pictar.bio.nyu.edu/), and miRanda (http://cbio.mskcc.org/cgi-bin/mirnaviewer/mirnaviewer.pl).

### Luciferase reporter assay

To test the direct binding of miR-10a to the target gene BCL6, a luciferase reporter assay was performed as previously described(Chen et al., 2009). The entire 3′-untranslated region (3′-UTR) of human BCL6 was amplified using PCR with human genomic DNA as a template. The PCR products were inserted into the p-MIR-reporter plasmid (Ambion), and the insertion was confirmed to be correct via sequencing. To test the binding specificity, the sequences that interacted with the miR-10a seed sequence were mutated (from CAGGGTT to GTCCCAA), and the mutant BCL6 3′-UTR was inserted into an equivalent luciferase reporter. For luciferase reporter assays, HEK293T cells were cultured in 24-well plates, and each well was transfected with 1 µg of firefly luciferase reporter plasmid, 1 µg of a β-galactosidase (β-gal) expression plasmid (Ambion), and equal amounts (100 pmol) of pre-miR-10a, anti-miR-10a, or the scrambled negative control RNA using Lipofectamine 2000 (Invitrogen). The β-gal plasmid was used as a transfection control. 24h post-transfection, the cells were assayed using a luciferase assay kit (Promega, Madison, WI, USA).

### siRNA and plasmid construction interference assay

The siRNA sequence targeting human BCL6 was designed and synthesized by Genepharma (Shanghai, China). The scrambled siRNA (Genepharma, Shanghai, China) was included as a negative control. A mammalian expression plasmid (pReceiver-M02-BCL6) designed to specifically express the full-length open reading frame (ORF) of human BCL6 without the miR-10a-responsive 3′-UTR was purchased from GeneCopoeia (Germantown, MD, USA). An empty plasmid (pReceiver-M02) served as a negative control. The siRNA or overexpression plasmid was transfected into OCI-Ly7 cells using Lipofectamine2000 (Invitrogen) according to the manufacturer’s instructions. Total RNA or protein was isolated 24 h or 48 h after transfection. Quantitative RT-PCR and Western blotting assessed the BCL6 mRNA and protein expression levels.

### Protein isolation and Western blotting

All of the cells were rinsed with PBS (pH 7.4) and lysed in RIPA Lysis buffer (Beyotime, China) supplemented with a protease and phosphatase inhibitor cocktail (Thermo Scientific 78440) on ice for 45 min. The DLBCL and RLH tissues were frozen solid with liquid nitrogen, ground into a powder and lysed in RIPA Lysis buffer containing the protease and phosphatase inhibitor cocktail on ice for 30 min. When necessary, sonication was used to facilitate cell lysis. Cell lysates or tissue homogenates were centrifuged for 15 min (12,000 g, 4°C). The supernatant was collected, and the protein concentration was calculated using the Pierce BCA protein assay kit (Thermo Scientific, Rockford, IL, USA). The BCL6 protein levels were analyzed using Western blotting with the corresponding antibodies. The protein levels were normalized by probing the same blots with GAPDH antibody. Experiments were conducted at least in independent triplicates. Densitometry analysis was performed using ImageJ, and normalized by the GAPDH intensity. The antibodies were purchased from the following sources: anti-BCL6 (sc-7388; Santa Cruz Biotechnology, CA, USA) and anti-GAPDH (sc-365062, Santa Cruz Biotechnology, CA, USA).

### Cell cycle assay

To analyze the cell cycle, OCI-LY7 cells were seeded in 6-well plates and transfected with pre-miR-10a, anti-miR-10a, BCL6 siRNA, or the BCL6 overexpression plasmid. Pre-miR-control, anti-miR-control, control siRNA, and control plasmid served as negative controls. Twenty-four hours after transfection, the cells were washed twice with PBS and fixed in 70% ethanol at 4°C overnight. Then, cells were resuspended in 1×PBS with 0.1% Triton X-100, 250 µg/mL RNase A for 1 h at 37°C. Staining for DNA content was performed using 50 mg/mL propidium iodide (BD Biosciences, San Jose, CA). The flow cytometry data was acquired on at least 10,000 cells from each sample on the Becton Dickinson FACS Calibur machine by the Cell Quest Pro software. For the cell cycle analysis, the Dean/Jett/Fox method of Flow Jo software was used.

### Apoptosis assays

The apoptosis of OCI-LY7 cells was tested using an Annexin V-FITC/propidium iodide (PI) staining assay. OCI-LY7 cells were cultured in 12-well plates and transfected with pre-miR-10a, anti-miR-10a, BCL6 siRNA, or the BCL6 overexpression plasmid to induce apoptosis. Pre-miR-control, anti-miR-control, control siRNA, and control plasmid served as negative controls. Cells were cultured overnight with serum-depleted medium, and then the cells were harvested. We detect the OCI-LY7 cells apoptosis of under normal or serum deprivation over night (Fig. S5). Flow cytometry analysis of apoptotic cells was performed using an Annexin V-FITC/PI staining kit (BD Biosciences, CA, USA). After washing with cold PBS, the cells were resuspended in binding buffer (100 mM HEPES at pH 7.4, 100 mmol/L NaCl, and 25 mmol/L CaCl_2_), followed by staining with Annexin V-FITC/PI at room temperature in the dark for 15 min. Apoptotic cells were then evaluated by gating PI- and Annexin V-positive cells using a fluorescence-activated cell-sorting (FACS) flow cytometer (BD Biosciences, San Jose, CA).

### Cell proliferation assay

OCI-LY7 cells were cultured in 6-well plates and transfected as mentioned above. Six hours after transfection, the cells were seeded into 96-well plates at a density of 2 × 10^4^ cells per well and then incubated overnight in IMDM supplemented with 10% FBS. Proliferation rates were determined at 12, 24, 36, 48 and 60 h after transfection. Cells were incubated with 10% CCK-8 (CK04-500, Dojindo) at 37°C for 3 h, and then the absorbance of each well was measured at a wave length of 450 nm. The day of transfection was set as Day 0, and the relative cell number was calculated based on the ratio of the absorbance at Day n to that at Day 0.

### Statistical analysis

All of the Western blotting images are representative of at least three independent experiments. Quantitative RT-PCR, the luciferase reporter assay, the cell cycle, cell proliferation assay and apoptosis assays were performed in triplicate, and each experiment were repeated three times. Data (mean ± SEM) are representative of at least three independent experiments. The numerical data were statistically analyzed by 2-tailed Student’s *t*-test. Bivariate correlation between two independent variables was calculated by Spearman’s rank correlation coefficient. Statistically significance was defined as *P* < 0.05.

## Electronic supplementary material

Below is the link to the electronic supplementary material.

Supplementary material 1 (PDF 297 kb)
