# Supplementary material for: miR-10a inhibits cell proliferation and promotes cell apoptosis by targeting BCL6 in diffuse large B-cell lymphoma
Source: Protein Cell. 2016 Nov 4;7(12):899–912. doi: 10.1007/s13238-016-0316-z (PMC5205661; doi:10.1007/s13238-016-0316-z)
Supplement: Supplementary file 1 — Supplementary material 1 (PDF 297 kb) [file 13238_2016_316_MOESM1_ESM.pdf]

miR-10a inhibits cell proliferation and promotes cell apoptosis by targeting BCL6 in diffuse large B-cell lymphoma

Qian Fan[1,*], Xiangrui Meng[1,*], Hongwei Liang[2,*], Huilaizhang[1,*],Xianming Liu[1,], Lanfang Li[1], Wei Li[1], Wu Sun[3], Haiyang Zhang[3], Ke Zen[2], Chen-Yu Zhang[2], Zhen Zhou[2,#], Xi Chen[2,#] and Yi Ba[3,#]

[1]Department of Lymphoma, Sino-US Center for Lymphoma and Leukemia, Tianjin Medical University Cancer Institute and Hospital, National Clinical Research Center of Cancer, Key Laboratory of Cancer Prevention and Therapy, Tianjin, 300060, China [2]State Key Laboratory of Pharmaceutical Biotechnology, NJU Advanced Institute of Life Sciences, Jiangsu Engineering Research Center for MicroRNA Biology and Biotechnology, School of Life Sciences, Nanjing University, Nanjing 210093, China; [3]Department of digestion, Tianjin Medical University Cancer Institute and Hospital, National Clinical Research Center of Cancer, Key Laboratory of Cancer Prevention and Therapy, Tianjin, 300060, China

Supplementary Table 1.

Clinical features of diffuse large B cell lymphoma patients.

Case No.	Age	Gender	Stage	Tumor type
Case1#	65	Male	II	Non-GCB
Case2#	38	Male	I	GCB
Case3#	62	Female	III	Non-GCB
Case4#	62	Female	II	Non-GCB
Case5#	59	Female	IV	Non-GCB
Case6#	43	Male	II	GCB
Case7#	60	Female	II	GCB
Case8#	57	Female	I	GCB
Case9#	45	Male	II	GCB

Supplementary Figure 1.

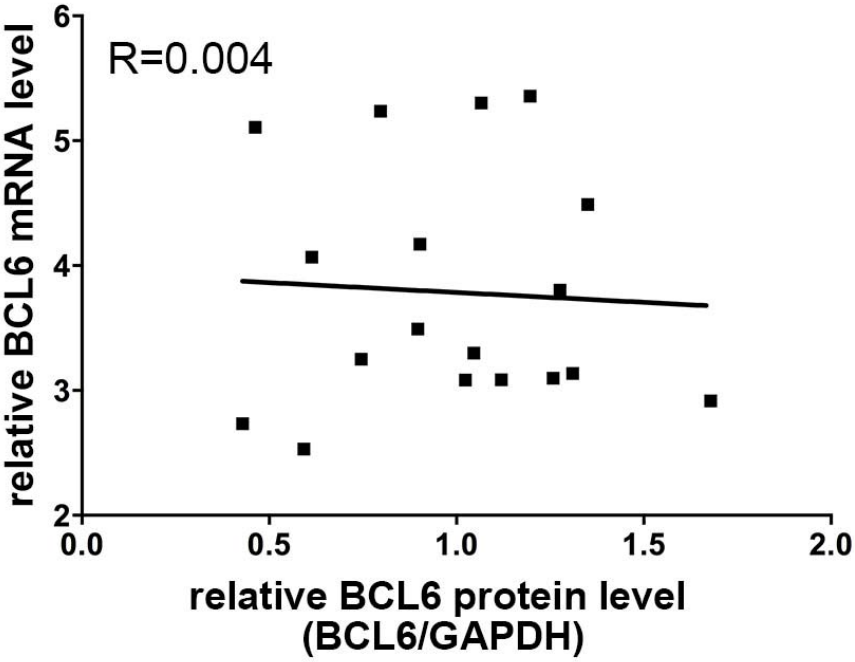

Pearson’s correlation scatter plot of the fold-change in the levels of BCL6 mRNA and BCL6 protein in the same DLBCL and RLH tissues.

Supplementary Figure 2.

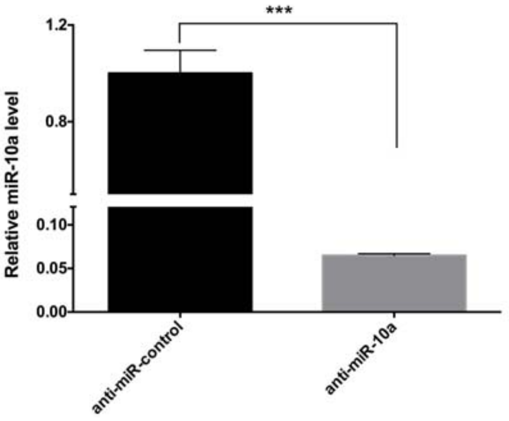

Quantitative RT-PCR analysis of miR-10a levels in 293T cells treated with equal dose of anti-miR-control or anti-miR-10a. (U6 snRNA was used as an internal control, and the relative amount of miRNA normalized to the U6 snRNA levels was calculated using the $2^{-\Delta\Delta CT}$ formula, in which $\Delta\Delta C_T = (C_{T\ miRNA} - C_{T\ U6})_{target} - (C_{T\ miRNA} - C_{T\ U6})_{control}$.) Data are the mean±SEM of 3 independent experiments performed in triplicate，*** $P < 0.001$

Supplementary Figure 3.

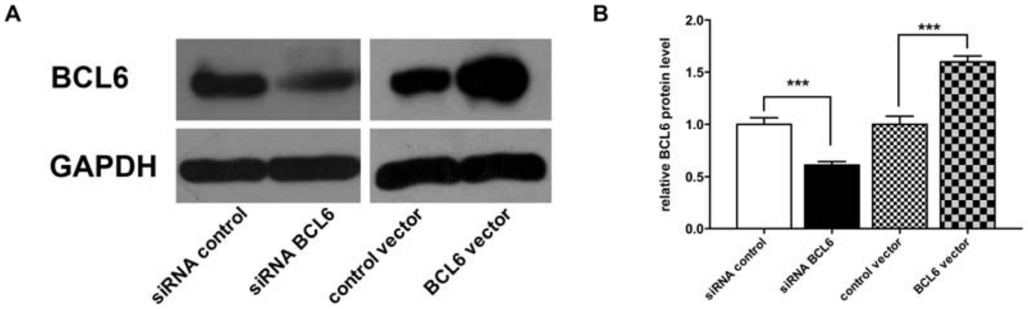

Western blot analysis of BCL6 protein levels in OCI-LY7 cells treated with control siRNA, BCL6 siRNA, control vector or a BCL6 overexpression vector; (A) representative image, (B) quantitative analysis. Data are the mean±SEM of 3 independent experiments performed in triplicate, *** $P < 0.001$.

Supplementary Figure 4.

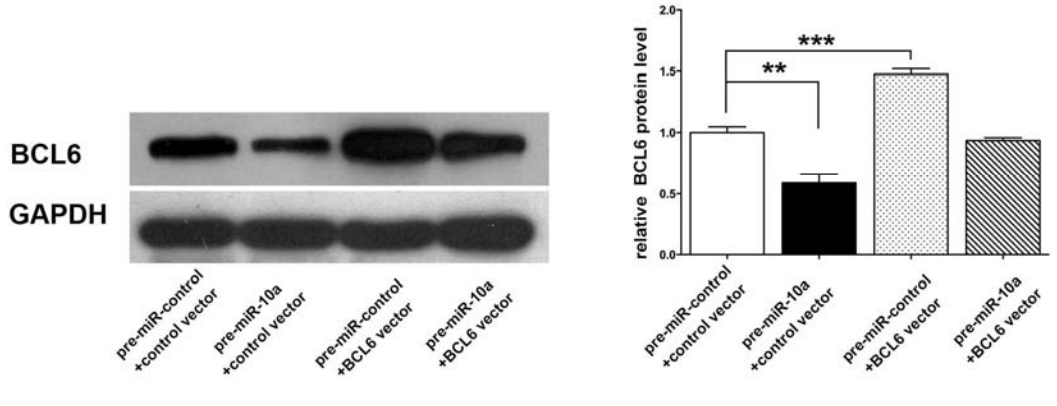

Western blot analysis of BCL6 protein levels in OCI-LY7 cells treated with pre-miR-control + control vector, pr-miR-10a + control vector, pre-miR-control +BCL6 vector, pre-miR-10a + BCL6 vector. Left panel: representative image; Right panel: quantitative analysis. Data are the mean±SEM of 3 independent experiments performed in triplicate，*** P < 0.001

Supplementary Figure 5.

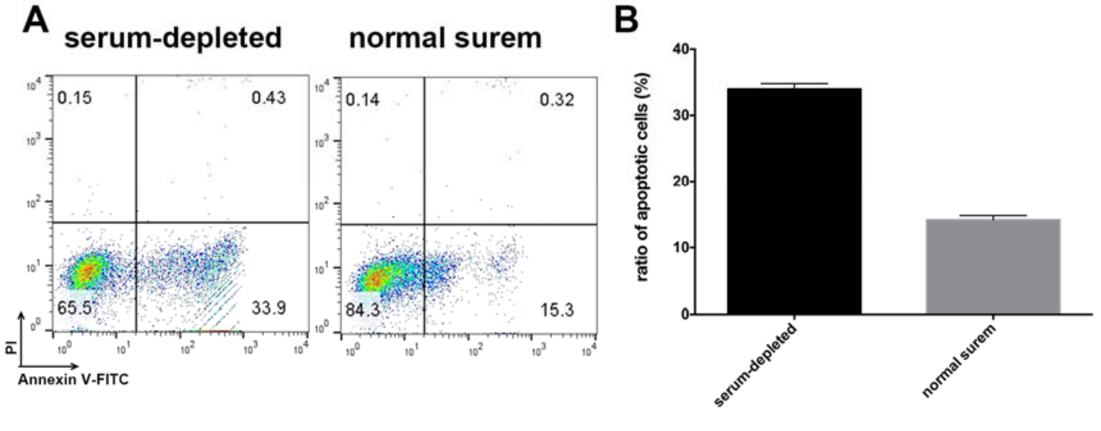

Annexin V/PI binding by flow cytometry was performed to detect the OCI-LY7 cells apoptosis of under normal or serum deprivation over night. A: representative image; B:

ratio of apoptotic OCI-LY7 cells.
